# Aggressive Myeloid Sarcoma Mimicking Burkitt's Lymphoma

**DOI:** 10.1002/jha2.70089

**Published:** 2025-06-26

**Authors:** Eman Hassan, Syed Aaquil Hasan, Samer Abbas, Peter Dyer, Hayder Hussein

**Affiliations:** ^1^ Department of Cardiovascular Sciences College of Medicine and Health University of Birmingham Birmingham UK; ^2^ Department of Haematology Queen Elizabeth Hospital Birmingham UK; ^3^ Department of Haematology University Hospitals of North Midlands Stoke‐on‐Trent UK

1

A 68‐year‐old female presented with a two‐month history of weight loss and back pain. Apart from mild anaemia (haemoglobin 9.8 g/dL) and high lactate dehydrogenase (LDH) 1618 U/L, all other blood investigations were otherwise normal. An initial CT scan of the abdomen and pelvis revealed extensive para‐aortic, and mesenteric lymphadenopathy (Figure 1). A biopsy of the para‐aortic lymph node demonstrated poorly differentiated malignancy. The initial panel of immunohistochemical markers was negative for tumour characterization. Further immunohistochemistry revealed CD117 and CD43 positivity in the tumour cells, raising the possibility of myeloid differentiation. However, a myeloid malignancy was considered unlikely due to the absence of expression of myeloperoxidase (MPO) and common leucocytic antigen (LCA), CD68, terminal deoxynucleotidyl transferase (TdT), CD15 or CD56.

**FIGURE 1:**
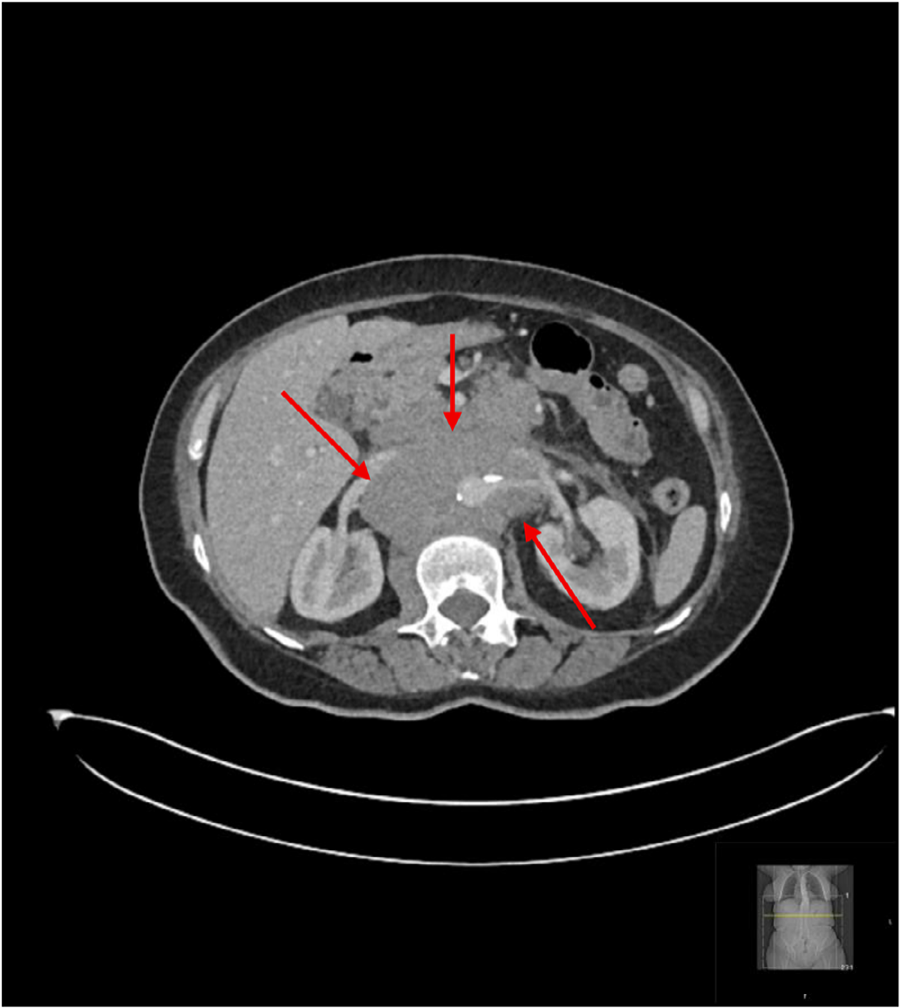
CT scan of the abdomen showing retroperitoneal lymphadenopathy (red arrows).

The absence of definitive lineage markers raised suspicion for a high‐grade, aggressive malignancy. However, due to the inconclusive immunophenotype, treatment was deferred, and a repeat biopsy was planned. A total of 5 weeks later, while awaiting further immunohistochemistry, the patient's condition deteriorated with worsening abdominal pain. A repeat CT scan revealed (Figure 2) new findings of ascites, a gallbladder mass, bilateral hydronephrosis and a moderate‐sized right pleural effusion. Cytospin of pleural fluid showed large vacuolated blasts with a morphology resembling Burkitt‐like cells (Figure 3). Flow cytometry of the blasts revealed CD45 negativity, raising suspicion for a non‐haematological malignancy. A screen for abnormal B or T lymphoid populations was negative.

**FIGURE 2:**
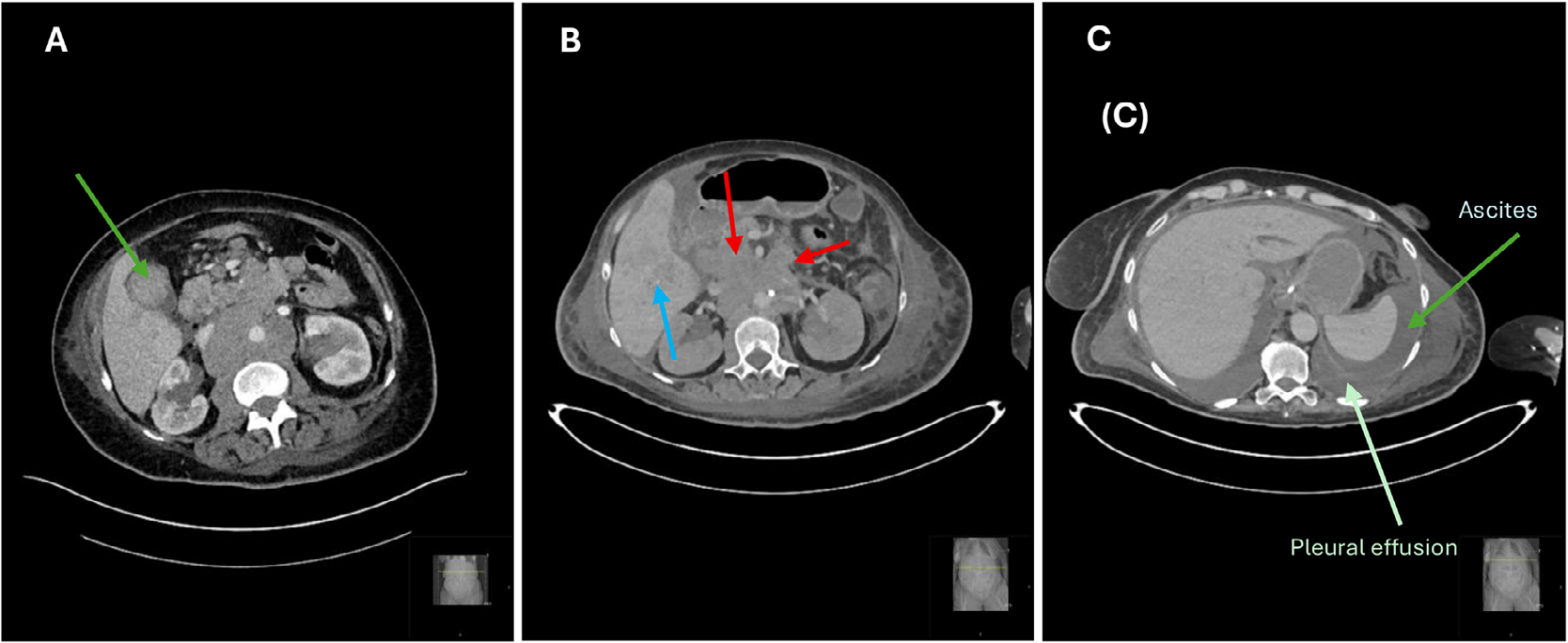
(A) Follow‐up CT scan showing a solid mass replacing the gallbladder (green arrow). (B) Axial CT showing multiple new subcentimeter low‐attenuation lesions throughout the right hepatic lobe (blue arrow). Findings are concerning for metastatic liver involvement. Progressive retroperitoneal lymphadenopathy (red arrows). (C) Axial CT image showing ascites and bilateral pleural effusions.

**FIGURE 3:**
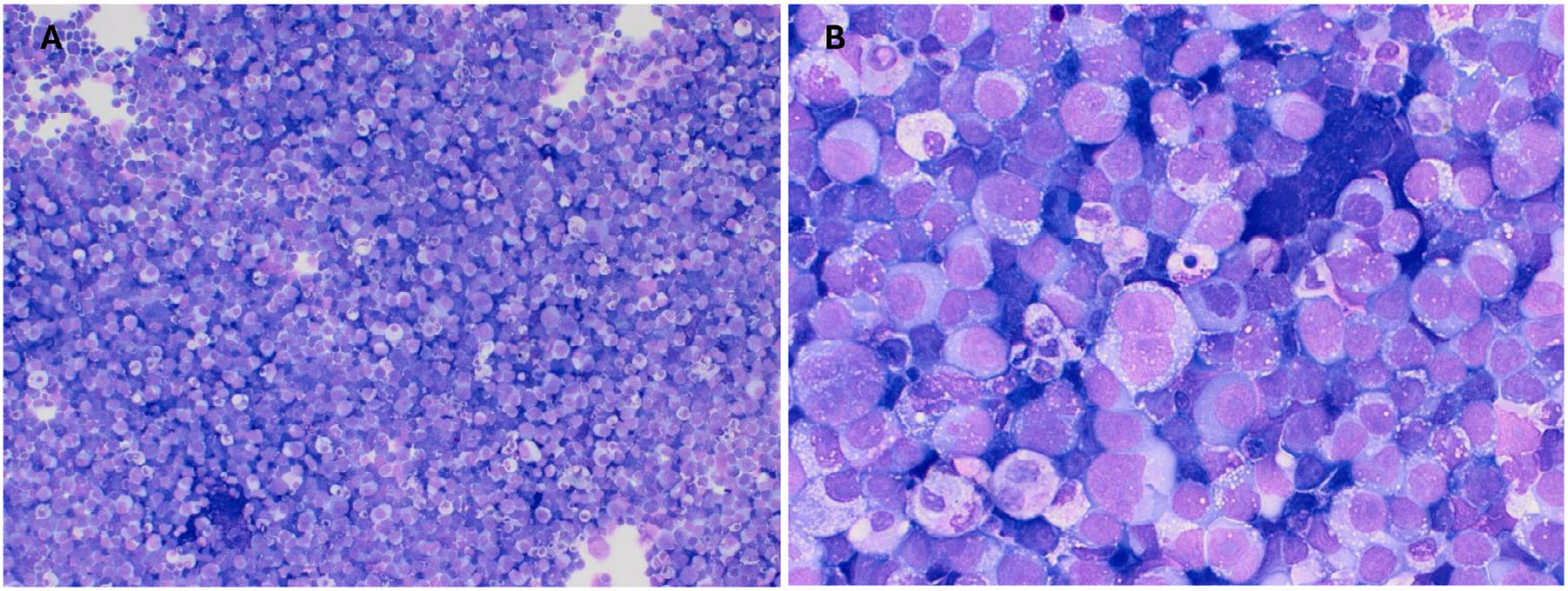
(A) Low‐power view (×100, May–Grünwald–Giemsa stain) of pleural fluid cytology showing a dense population of large, immature monomorphic cells with high nuclear‐to‐cytoplasmic ratio. (B) High‐power view (×400, May– Grünwald–Giemsa stain) reveals large pleomorphic blastoid cells with irregularly shaped (round to ovoid) nuclei, open chromatin, 1–2 prominent nucleoli, and abundant basophilic cytoplasm containing multiple fine cytoplasmic vacuoles.

An acute myeloid leukaemia (AML) panel showed ∼97.6% myeloid cells (CD33+ and/or CD13+) with reduced expression of CD11b+. 5.3% of cells were CD14+, and 66% of blasts were CD45 negative, CD34+, CD117+, HLA‐DR weak, CD38+ and CLL1+. This blast population was negative for CD3, CD4, CD7, CD19, CD30, CD56, CD123, MPO, cytoplasmic CD3, CD22 and CD79a. These findings were consistent with a diagnosis of AML. Blastic plasmacytoid dendritic cell neoplasm and systemic mastocytosis were considered; however, the absence of CD123 expression and lack of morphological features of mastocytosis made these diagnoses unlikely. Molecular studies were not performed on the available samples, and obtaining a new sample was deemed impractical due to the patient's clinical deterioration. The bone marrow aspirate findings showed no evidence of malignancy, and the diagnosis of myeloid sarcoma was subsequently established. The patient started on intensive chemotherapy. However, within days of treatment, she developed tumour lysis syndrome, which required haemodialysis. Unfortunately, the patient deteriorated further due to sepsis and passed away shortly after commencing treatment. This case highlights the diagnostic complexity of myeloid sarcoma, which can mimic lymphoma in its clinical and morphological presentation.

## Author Contributions

All authors contributed equally to the acquisition of clinical data, image collection, literature review, and drafting of the manuscript. All authors reviewed and approved the final version of the manuscript.

## Ethics Statement

This is a retrospective case report with no identifiable patient data.

## Consent

Written informed consent was obtained from the patient's next of kin for publication of this case report and accompanying images.

## Conflicts of Interest

The authors declare no conflicts of interest.

## Data Availability

No datasets were generated or analysed during the current study.

